# Computational Probing of Tin-Based Lead-Free Perovskite Solar Cells: Effects of Absorber Parameters and Various Electron Transport Layer Materials on Device Performance

**DOI:** 10.3390/ma15217859

**Published:** 2022-11-07

**Authors:** Arunkumar Prabhakaran Shyma, Raja Sellappan

**Affiliations:** 1 Department of Physics, School of Advanced Sciences, Vellore Institute of Technology, Vellore 632014, India; 2 Centre for Nanotechnology Research, Vellore Institute of Technology, Vellore 632014, India

**Keywords:** tin-based perovskite solar cell, lead toxicity, numerical analysis, absorber layer, quantum efficiency, defect density, ETL materials

## Abstract

Tin-based perovskite solar cells have gained global research attention due to the lead toxicity and health risk associated with its lead-based analog. The promising opto-electrical properties of the Tin-based perovskite have attracted researchers to work on developing Tin-based perovskite solar cells with higher efficiencies comparable to lead-based analogs. Tin-based perovskites outperform lead-based ones in areas such as optimal band gap and carrier mobility. A detailed understanding of the effects of each parameter and working conditions on Tin-based perovskite is crucial in order to improve efficiency. In the present work, we have carried out a numerical simulation of a planar heterojunction Tin-based (CH_3_NH_3_SnI_3_) perovskite solar cell employing a SCAPS 1D simulator. Device parameters, namely, the thickness of the absorber layer, the defect density of the absorber layer, working temperature, series resistance, and metalwork function, were exclusively investigated. ZnO was employed as the ETL (electron transport layer) material in the initial simulation to obtain optimized parameters and attained a maximum efficiency of 19.62% with 1.1089 V open circuit potential (V_oc_) at 700 nm thickness (absorber layer). Further, different ETL materials were introduced into the optimized device architecture, and the Zn_2_SnO_4_-based device delivered an efficiency of 24.3% with a V_oc_ of 1.1857 V. The obtained results indicate a strong possibility to model and construct better-performing perovskite solar cells based on Tin (Sn) with Zn_2_SnO_4_ as the ETL layer.

## 1. Introduction

In recent times, perovskite solar cells based on organometal halides have gained a significant amount of attention due to their opto-electrical properties and low manufacturing costs. The quantum leap in terms of efficiency in perovskite-based solar cells (PSCs) from 3.8% to 25.2% makes it further intriguing yet challenging [1,2,3,4,5,6,7]. This remarkable performance of PSCs can be ascribed to the distinctive ABX_3_ crystal structure of perovskite that consists of a monovalent organic or inorganic cation (A), divalent cation (B), and monovalent anion (X), which delivers exceptional photovoltaic characteristics including intense light absorption, ample ambipolar charge mobility, and minimal exciton binding energy (<25 meV) [8,9].

On the other hand, the main charm behind the performance of organometal halide perovskite is attributed to the role played by Pb [10]. Even though Pb-based perovskite solar cells lose their charm when coming to the water solubility of lead which leads to severe health risks and environmental issues [11]. The World Health Organization (WHO) has recently enlisted Pb as one of the ten most toxic materials with respect to human health and initiated strict measures to lessen the usage of Pb [12]. The health hazards regarding the usage of Pb drove the global research community to explore lead-free perovskite structures. Elements that possess a + 2 oxidation state are considered viable alternatives for lead-free halide perovskite structure. A large number of materials can be considered in this manner. Many materials were tried and found not to implement in the perovskite structure since they possess large band gaps (Be^2+^, Ca^2+^, Sr^2+^, Ba^2+^) and toxicity (Cd^2+^, Hg^2+^) [13]. Sn-based perovskites are found to be the best alternative to Pb since it possesses an optical bandgap in the range of 1.2 to 1.5 eV, which satisfies the Shockley–Queisser Limit (~1.3 eV). This ideal band gap expedited the development of Pb-free Sn-based perovskite solar cells [14,15]. The low bimolecular constant to mobility ratio of Sn-based perovskites also provides the basis of replacement since it exhibits a similar ratio with the Pb-containing counterparts [16]. The comparability of Sn-based perovskites to the Pb-containing counterparts has gained significant research attention in the field of perovskite solar cells as a viable alternative for the future.

Recently, many efforts have been made to improve the performance of Pb-free perovskite solar cells, such as compositional and band gap engineering. In spite of the developments in performance enhancements, the overall efficiency (PCE) of the device is limited due to external as well as internal parameters. In this scenario, the simulation of these devices is crucial to understand the correlation between device parameters and cell performance. The main motive behind this present research work is to simulate and optimize the device parameters with different electron transport layers (ETLs) for the optimum efficiency of Pb-free perovskite solar cells. Among the various metal oxides, Zn_2_SnO_4_-based ETL is a robust and promising option due to its excellent electronic properties, such as a wide band gap (3.35 eV) [17] and a relatively low refractive index of 2 [18]. Zn_2_SnO_4-_based ETL is expected to showcase fast electron diffusion and charge injection, which provides enhanced light harvesting and charge collection efficiency for the solar cell [19]. Recently, Wu et al. demonstrated an MAPbI_3_-based perovskite solar cell with Zn_2_SnO_4_ as an ETL and managed to obtain an efficiency of 16.38% under AM 1.5G irradiation [20]. ETLs such as Zn_2_SnO_4_ are potential candidates to elevate the output of underperforming toxic-free MASnI_3_-based perovskite solar cells over toxic lead-based MAPbI_3-_based ones.

In this work, the SCAPS 1D simulator is employed to examine the different device parameters such as absorber layer thickness, working temperature, quantum efficiency, series resistance, etc. SCAPS 1D is a numerical simulation program developed at the Department of Electronics and Information Systems of the University of Gent, Belgium. The basic equations employed in the numerical simulation are Poisson’s equation (Equation (1)), hole (Equation (2)), and electron (Equation (3)) continuity equations as follows.

(1) $$\frac{d}{dx}(–\varepsilon (x)\frac{d}{dx})\;=\;q[p\left(x\right)-n\left(x\right)+N_{d}^{+}\left(x\right)-N_{a}^{-}\left(x\right)\;+\;p_{t}(x)-\;n_{t}(x)]$$

(2) $$\frac{dp_{n}}{dt}=G_{p}-\frac{p_{n}-p_{no}}{\tau_{p}}-\;p_{n}\mu_{p}\frac{d_{\xi }}{d_{x}}\;-\mu_{p}\xi_{p}\frac{dp_{n}}{dx}+D_{p}\frac{d^{2}p_{n}}{dx^{2}}\;$$

(3) $$\frac{dn_{p}}{dt}=\;G_{n}-\frac{n_{p}-n_{po}}{\tau_{n}}\;-\;n_{p}\mu_{n}\frac{\xi d}{dx}\;-\mu_{n}\xi_{n}\frac{dn_{p}}{dx}\;+D_{n}\frac{d^{2}n_{p}}{dx^{2}}\;$$

Here, *D* stands for the diffusion coefficient, *Ψ* represents electrostatic potential, *G* defines the generation rate, and *ξ* is the electric field and *ε* dielectric permittivity. *p, n, p_t_, n_t_* represents free holes, free electrons, trapped holes, and trapped electrons, respectively. Here electrostatic potential is the function of hole and electron concentration, as shown in Equations (2) and (3). $N_{d}^{+}$ and $N_{a}^{-}$ are stands for ionized acceptor doping concentration as well as ionized donor concentration, respectively [21,22,23,24,25]. A recombination rate of 1.006 × 10^22^ cm^−3^ s^−1^ is observed for a defect density of 10^15^ cm^−3^, in which the particular defect density can be considered as the optimized one for further device simulation.

## 2. Device Modelling and Simulation

**Figure 1:**
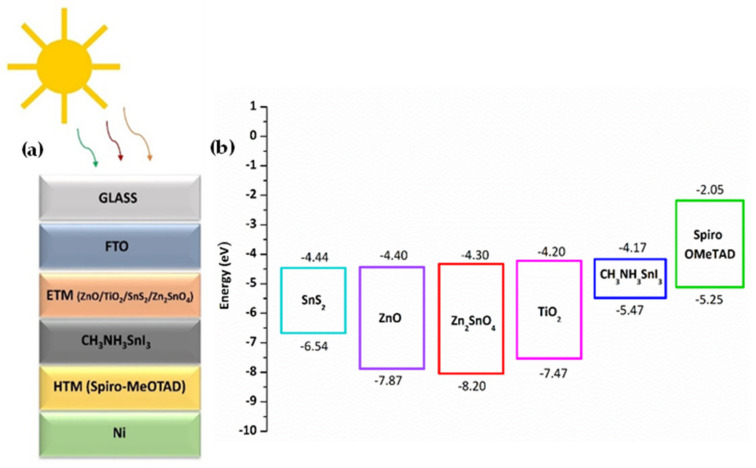
(**a**) Device architecture of the Sn based perovskite solar cell and (**b**) Energy band alignment for the proposed devices.

Currently, research community has shown a significant amount of interest in theoretical studies and modeling of perovskite solar cells [26,27,28,29]. The device architecture and energy band diagram of the proposed perovskite solar cell is depicted in Figure 1 [30,31,32,33]. Herein, we have proposed a Pb-free Sn-based perovskite solar cell, which consists of five main layers. ZnO/TiO_2_/SnS_2_/Zn_2_SnO_4_, Methylammonium tin triiodide (CH_3_NH_3_SnI_3_), and Spiro-OMeTAD were employed as electron transport layer (ETL), Absorber layer, and hole transporting layer (HTL), respectively to acquire the optimum parameters for the device. ZnO was used for initial simulation to obtain optimized parameters for the device. Further, a detailed comparison between different ETL materials was also conducted to find the best-suited ETL for Pb-free perovskite solar cell. An n-i-p planar architecture was considered in which the absorber layer is sandwiched between ETL and HTM. Thermal velocities of hole and electron were kept at 1 × 10^7^ cm s^−1^. All the simulations were conducted under an air mass of AM 1.5G and an illumination of 1000 W m^−2^. The operating temperature was varied from 300 to 370 K in order to obtain the optimum working conditions. Device and material parameters that are used for the simulation are acquired from previous literature and theories [30,31,32,33]. The parameters employed in the simulation are summarized in Table 1 and Table 2. We have separately carried out the simulation for each metal back contact to find the best performance. Among the simulated results, device employed with Ni and Pt has exhibited better power conversion efficiency compared to other candidates. We have fixed Ni as the back contact, considering the cost-effectiveness of Ni compared to Pt. The simulated device performance for each back contact is given in Table 3. We have tuned and further enhanced the device performance employing the optimum values of various parameters obtained through numerical simulation, such as open circuit voltage (V_oc_), Short circuit current density (J_sc_), Fill factor, and power conversion efficiency (PCE).

**Figure 2:**
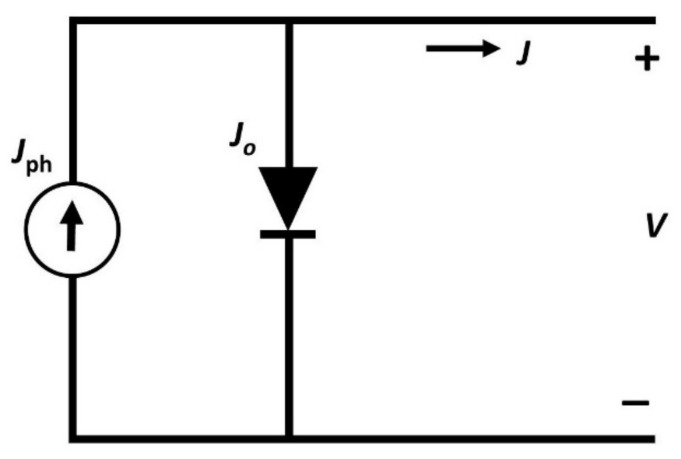
Equivalent electrical circuit diagram for a solar cell under illumination.

The equivalent circuit of solar cell under light illumination is shown in Figure 2. The perovskite absorber layer generates electron and hole pairs once the light is illuminated and develops a voltage across the device. A current is produced within the device as a result of the movement of electrons and holes. The generated current is directly proportional to the illumination rate and additionally depends on the size of the surface area being illuminated. Here, V_oc_ is also a function of illumination.

## 3. Results and Discussion

### 3.1. Effect of Absorber Layer Thickness on Device Performance

**Figure 3:**
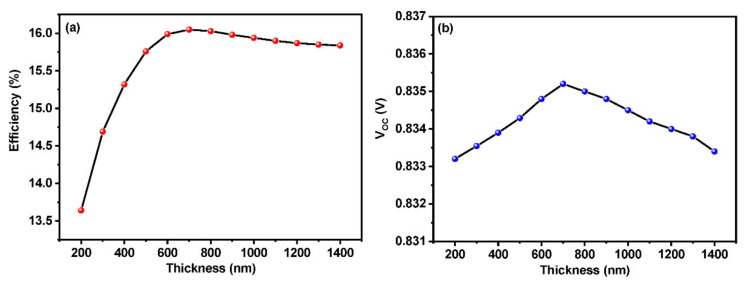
Effect of absorber layer thickness on (**a**) efficiency and (**b**) V_oc_.

**Figure 4:**
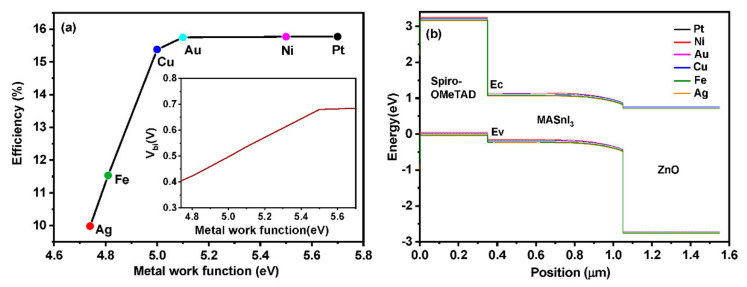
(**a**) Efficiency as a function of metal work function with varying back contacts and metal built in potential according to the metal contact (Inset). (**b**) Band diagram of constructed perovskite solar cell with different back contacts.

The thickness of the absorber layer plays a vital role in the efficiency of the perovskite solar cell. Response of the solar spectrum by a device can be described as a function of absorber layer thickness. In the present study, the thickness of the absorber layer (CH_3_NH_3_SnI_3_) has been varied from 100 to 1400 nanometers with spiro-OMeTAD as HTL and ZnO as ETL. The effects of thickness on the efficiency and open circuit potential (V_oc_) are shown in Figure 3a,b. The diffusion length of electrons and holes has a direct influence on the absorber layer thickness, i.e., the thickness of the absorber layer should be smaller than the diffusion length of electrons and holes [34]. It ensures that electrons and holes reach the electrodes for power generation. The light absorption rate increases as a consequence of the increment in the layer thickness, which leads to higher V_oc_, J_sc_, and photoconversion efficiency. On the flip side, the increase in the absorber layer thickness results in the reduction of the back contact recombination current density [35]. Figure 4a,b shows the effect of absorber layer thickness on the device parameters such as efficiency (PCE) and open circuit voltage (V_oc_), respectively. It is visible that the PCE and V_oc_ increase with the increase in the absorber layer thickness in the range of 200 to 700 nm. Beyond 700 nm, it could be seen that a decrement in the PCE as well as the V_oc_ as a result of the increase in the carrier diffusion length. Here, the photons are absorbed deep into the absorber layer limiting them from the depletion region. As a consequence, the generated electron hole pair leads to the bulk recombination instead of reaching space charge region during their life span [36,37]. The decline in V_oc_ right after the optimum thickness of the absorber layer can be best explained as the result of the higher recombination rate. Thus, the decline in V_oc_ also reflects in the efficiency as well. The V_oc_ can be expressed as

(4) $$V_{oc}=(\frac{nkT}{q})\;\ln\;({\frac{I_{L}}{I}}_{0}+1)\;$$

Here, n stands for diode ideality factor and *kT/q* refers to the thermal voltage. *I*_0_ and *I_L_* are dark and light saturation currents, respectively. A thin absorber layer comes with the advantage of minimal electron-hole recombination, which possesses a minimal value of dark saturation current. This might be the possible reason for the gradual increment of V_oc_ up to a certain thickness. After the optimum thickness of the perovskite absorber layer, V_oc_ decreases due to the increase in dark saturation current. We have also experienced a decrement in the fill factor with an increasing thickness which causes more internal power usage [34]. From the simulated results, it is clear that the selection of the absorber layer thickness has to be made according to the diffusion length of the charge carriers. The optimum thickness of the absorber layer should be lesser than the diffusion length in order to make sure the maximum possible efficiency. As per the study, the optimum thickness for the proposed absorber layer is 700 nm which delivers a PCE of 16.1%. A thicker absorber layer (between 600 and 800 nm) can absorb more photons, leading to higher efficiency utilizing AM 1.5 G condition. The obtained results are in line with the previous literature [27]. Additionally, the diffusion length with respect to absorber defect density has been depicted in Table 3. It can be seen that the minimal recombination rate corresponds to a defect density value of 10^15^ cm^−3^. The diffusion length that corresponds to the above defect density is around 750 nm which is comparable with the optimized absorber thickness of 700 nm. As mentioned earlier, the thickness of the absorber layer should not deviate too much from the diffusion length value, which will result in recombination and a decrement in efficiency.

### 3.2. Effect of Different Metal Back Contacts on Device Performance

Several back contact materials, such as platinum (Pt), nickel (Ni), gold (Au), copper (Cu), iron (Fe), and silver (Ag), are employed in the perovskite device architecture. Figure 4a shows the efficiency obtained by devices as a function of metal contact work function. A detailed simulation has been employed with different metal contacts, and output values are depicted in Table 4. The simulated results exhibit an increment in the efficiency for higher work function values.

However, the efficiency saturates right after a particular work function value. Most of the carrier barrier height decreases as a result of increased metal work function. Increasing work function also affects the band bending at the metal-semiconductor interface, which makes contact with the ohmic [30]. The band diagram (Figure 4b) shows that Ni, Pt, and Au are overlapped due to their work function, which lies in the range of 5.1 to 5.7 Ev [38]. Cu, Fe, and Ag exhibit different alignments due to their lower built-in potential (V_bi_). The built-in potential grows linearly up to 5.4 eV, and after that, it attains a saturated position. The built-in potential is considered to be the potential net drop across the absorber layer. This potential drop plays a major role by triggering an electric field towards the p-n junction. The existence of such potential is beneficial for the drift of charge carriers within the absorber layer. The electric field formed as the result of the built-in potential drifts the generated electron-hole pairs towards the junction by increasing the diffusion length and collection of photo-generated carriers at the n side. Thus, the high work function of the back contact enhances the built-in voltage and improves the efficiency of the device [39].

Lower work function leads to lower efficiency since the electric field near to HTM/back contact interface becomes negative due to the tendency of holes to travel toward the electrode [31]. Pt and Ni show higher performance as back contacts in the perovskite device architecture. The cost-effectiveness of Ni makes it a promising back metal contact for enhanced device performance when compared to Pt.

### 3.3. Effect of Density of States (DOS) on the Absorber Layer

**Figure 5:**
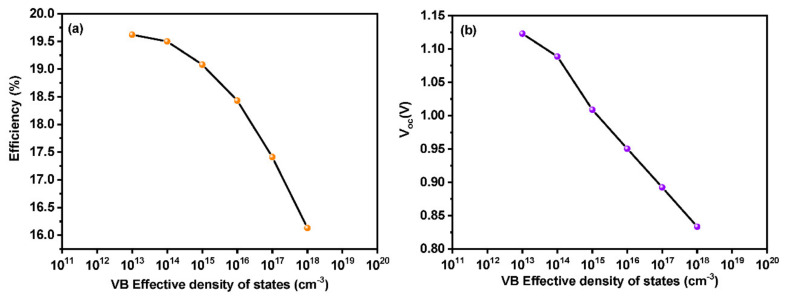
Effect of density of States on (**a**) efficiency and (**b**) V_oc_ of the device.

In order to find out the effect of the density of states of the perovskite absorber layer on the efficiency of the device, we have carried out a simulation of the device with varying density of states (N_V_) of the absorber layer ranging from 10^13^ to 10^19^ cm^−3^. Figure 5 depicts the efficiency as well as the V_oc_ of the device as a function of DOS. It is clear from Figure 5a that efficiency decreases with an increase in Nv of the perovskite absorber layer. The higher number of holes in the absorber layer leads it to participate in the reverse saturation current. Thus, the device suffers from poor electric conversion efficiency as a result of the voltage drop [40].

### 3.4. Effect of Temperature on the Device Performance

**Figure 6:**
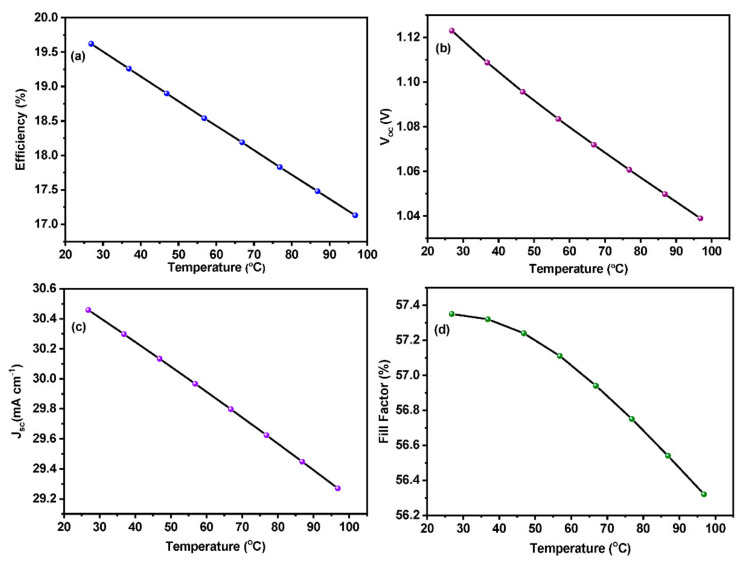
Effect of device temperature on (**a**) efficiency, (**b**) V_oc_ (**c**) J_sc_ and (**d**) Fill factor.

Solar cells are sensitive to temperature, like all other semiconductor devices. An increase in temperature decreases the band gap of the semiconductor, which affects most of the semiconductor parameters. The energy of the bound electron increases as a result of increasing the temperature. The difference between the rest state of the electrons and the excited state as a result of sunlight determines the potential difference that is a crucial parameter for perovskite solar cells. Since the electrons are already excited due to the increased heat energy, then the probability of electrons becoming excited as a result of sunlight is limited. The potential drop will be minimal due to the pre-excited electrons as a result of temperature leading to a low-performing device. Working temperature is a crucial factor when it comes to perovskite solar cells. Especially, parameters such as J_sc_ and V_oc_ are highly related to their working temperature [41]. An operating temperature of around 27 °C was employed for most of the device simulations. In order to probe the effect of temperature on Pb-free perovskite solar cells, the working temperature of the device was varied from 27 to 97 °C under constant illumination of 1000 W m^−^^2^. The equivalent circuit diagram shown in Figure 2 can be effectively used to draw a relation between device parameters and temperature [42]. Figure 6 shows the device parameters as a function of the temperature.

The overall efficiency of the device is significantly decreased as temperature increases. V_oc_ is a crucial factor that determines the performance of the device. As a result of the increase in device temperature, the reverse saturation current also increases exponentially, which leads to a reduction in the V_oc_ [43]. The PCE of the device also drastically decreases along with the V_oc_. As temperature increases, device parameters such as band gaps, electron and hole mobility, and carrier concentration are affected, which results in lower efficiency of the device [44,45]. From the obtained simulation results, we can conclude that the operating temperature exhibits a linear relationship with the device efficiency. Thus, the efficiency and the generated power output of the perovskite solar cell strongly adhere to the working temperature also.

### 3.5. Effect of Absorber Defect Density on Device Performance

**Figure 7:**
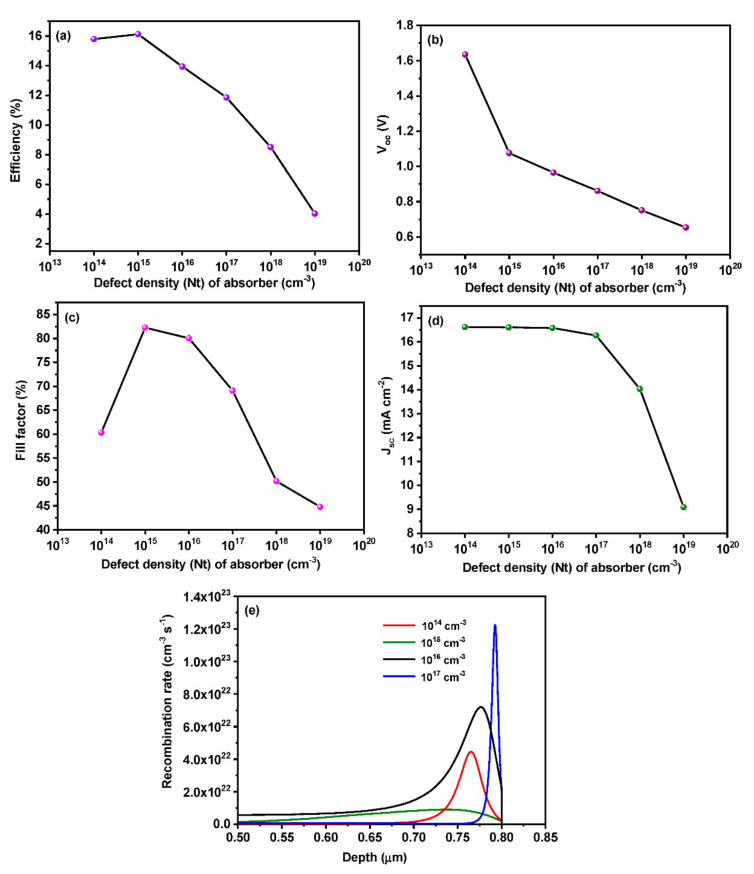
Effect of absorber layer defect density on (**a**) efficiency, (**b**) V_oc_, (**c**) fill factor and (**d**) J_sc_ (**e**) recombination rate inside perovskite.

The defect on the absorber layer must be well probed in order to obtain maximum efficiency out of the device. The capture cross-section of electrons and holes were kept as 10^−17^ and 10^−15^ cm^2^, respectively. Gaussian distribution was employed with an energy level of 0.70 eV [46,47]. We have varied the absorber defect density from 10^14^ to 10^20^ cm^−3^ to draw a relationship between defect density and efficiency, as shown in Figure 7. A drastic drop in efficiency can be observed with the increasing defect density of the absorber layer. The deep energy levels in the band gap act as Shockley–Read Hall non-radiative recombination centers since the photo-electrons are mainly generated from the absorber layer. As a consequence of these centers, the minority carrier lifetime becomes short, and charge recombination dominates over V_oc_ [27]. If the defect concentration exceeds the doping concentration of the absorber layer, the device loses its semi-conductivity, and the formation of the proper p-n junction is hindered. This affects the proper functioning of the device, and the efficiency decreases drastically [48]. According to Figure 7c, we can see a drop in fill factor from 60.31% to 44.79% with an increase in defect density from 10^14^ to 10^19^ cm ^−3^. A rapid increase in fill factor value can be seen between 10^14^ and 10^15^ cm^−3^ since the doping density of the perovskite layer was set as 10^15^ cm^−3^ which is in line with the defect density. Efficiency, V_oc_, and J_sc_ also exhibit a decrement as defect density increases. Consequently, all device parameters decrease with the increase in defect density values. Thus, in order to obtain an enhanced output from the device, the absorber layer must have high quality with a few defects. As shown in Figure 7e, decreasing the defect density value leads to an increase in carrier lifetime and diffusion length. Hence, less recombination is observed which leads to better performance.

### 3.6. Effect of Change in Series Resistance on Device Performance

**Figure 8:**
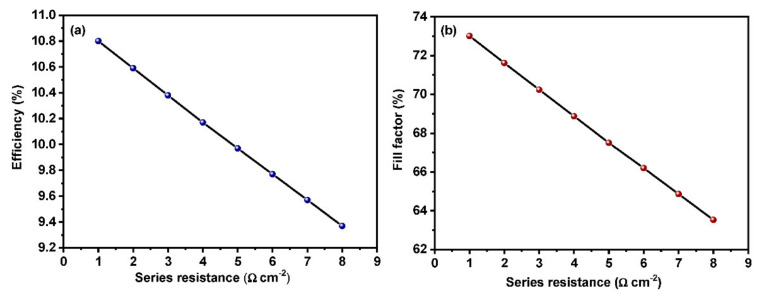
Effect of series resistance on (**a**) efficiency and (**b**) fill factor.

Series resistance is one of the important parameters when it comes to the performance of the device. It has a direct influence on the fill factor and J_sc_. Form Figure 8b, it is evident that the fill factor decreases with the increase in the series resistance. Thus, higher values of series resistance in perovskite solar cells result in poor conversion efficiency. The simulation has been carried out to study the effect of series resistance on PCE and fill factor of perovskite solar cells. The equivalent circuit for a basic solar cell is given in Figure 2.

(5) $$I_{sc}=I_{0}{\;}_{(}e^{V_{oc}q/nKT}-1)$$

(6) $$Isc\;=\;I_{L}\;-\;I_{0}\;\left(e^{V_{oc}q/nKT}-1\right)\;-\;\frac{V_{oc}+I_{sc\;}r_{s}}{r_{sh}}\;$$

Here, Equation (5) can be used to analyze the effect of series resistance on device performance [49] and Equation (6) stands for the equivalent circuit. Here *I_L_* and *I_sc_* are light-induced and short circuit currents, respectively, and *r_sh_* stands for shunt resistance. It is evident from the above equation that the value of *I_sc_* decreases with an increase in series resistance *(r_s_*). This acts as the main reason for the decrement in efficiency as well as the fill factor. At lower resistance values, the device exhibits an enhanced performance with a higher fill factor value (Figure 8b). A significant drop in the efficiency of the device can be seen in Figure 8a as a function of the increase in series resistance. The results obtained from the study are in line with previously reported literature [50].

### 3.7. Optimized Device Performance

**Figure 9:**
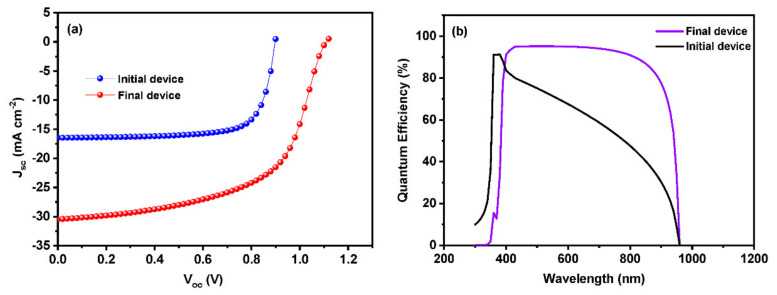
(**a**) J-V curves and (**b**) external quantum efficiency of initial and final device based on ZnO ETL.

The final device has been simulated employing all the optimized parameters, namely, the thickness of the absorber layer, absorber layer defect density, working temperature, series resistance, metalwork function, etc. The performance of the initial and final devices is shown in Figure 9. The initial and final devices obtained an efficiency of 11.0 and 19.62%. The final device delivered a short circuit current density of 30.45 mA cm^−2^, which is 1.8 fold higher than the initial one. A significant increase in the case of V_oc_ can be seen in the final device compared to the initial one. A decrement in the case of the fill factor has occurred from 74 to 58 in the case of the final device, which might be attributed to the increase in short circuit current density. The V_oc_ obtained for the initial and final devices are 0.8985 and 1.1089 V, respectively. Around 20% increment had happened in V_oc_ for the final device while comparing to the initial device. The final optimized device exhibited 56.3% higher efficiency than the initial one, which also shows the potential ability of Sn-based perovskite solar cells compared to their Pb-based counterparts. Figure 9b shows the external quantum efficiency of the initial and final devices. The final device exhibits desirable output throughout the visible as well as the near-infrared region compared to the initial device.

### 3.8. Performance of Different ETL Materials in the Optimized Device Architecture

**Figure 10:**
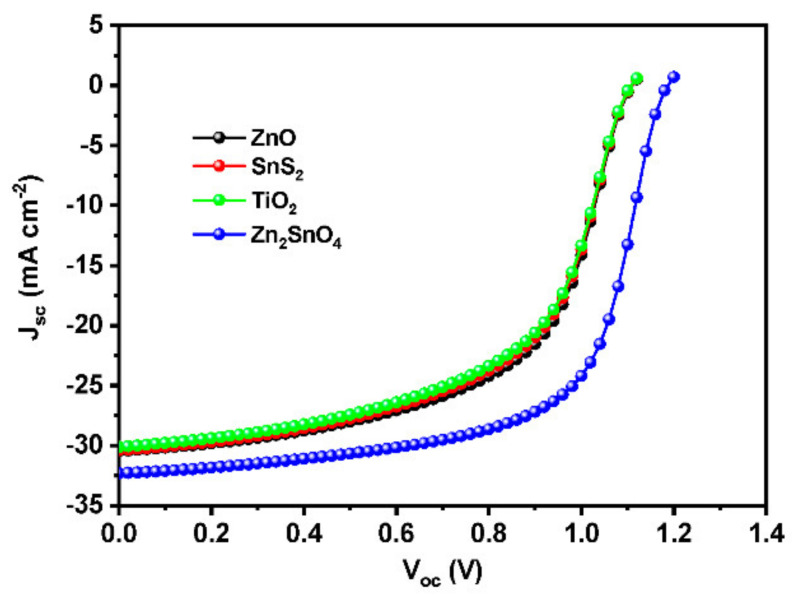
J-V curves for different ETL materials.

Different ETL materials were introduced into the device architecture after optimizing the device parameters, such as thickness and defect density of the absorber layer, device temperature, resistance, recombination rate, etc., with ZnO as ETL. We were able to identify Zn_2_SnO_4_ as a viable and effective ETL material for lead-free perovskite solar cells among the different ETL candidates implemented, such as SnS_2_, ZnO, TiO_2_, etc (Figure 10). The Zn_2_SnO_4-_based device obtained an efficiency of 24.73%, which is comparable with previous works [20,51] with an enhanced V_oc_ of 1.1857 V. The high electron affinity of Zn_2_SnO_4_ could be the reason for the enhanced performance of the cell [52]. The detailed performance of all ETL candidates has depicted in Table 5.

## 4. Conclusions

Pb-free Sn-based (CH_3_NH_3_SnI_3_) perovskite solar cells have been designed and constructed using SCAPS. The designed model was verified by comparing the parameters with the reported literature. Different absorber parameters, along with the working conditions of the device, have been exclusively studied. The thickness and defect density of the absorber layer varied from 100 to 1400 nm and 10^14^ to 10^17^ cm^−3^, respectively. Increasing the absorber layer thickness also induced the J_sc_ value. An absorber layer thickness of 700 nm can deliver good efficiency of 19.68%. Further, the bulk defect also affected the overall performance of the device. When the bulk defect of the absorber layer was increased, all device parameters tended to decrease drastically. This might be due to the higher recombination rate and subsequent increase in the series resistance. The comparable or higher bulk defect with respect to the doping concentration of the absorber layer can also be a reason for the drop in device parameters. The rise in series resistance, as well as working temperature, reduced the device’s performance. A suitable absorber layer thickness of 700 nm and defect density of 10^15^ cm^−3^ was found to be optimal. The Zn_2_SnO_4-_based device delivered an efficiency of 24.73%, J_sc_ of 32.30 mA cm^−2^, V_oc_ of 1.1857 V, and FF of 64.58%. The enhanced performance of Zn_2_SnO_4_ as an ETL material might be due to the higher electron affinity, which transports a greater number of effective electrons from the absorber layer. Further, Extensive experimental studies are required for the investigation of the proposed Sn-based perovskite solar cell in order to completely replace the lead-based analogues since lead toxicity is a serious threat to our ecosystem.

## Figures and Tables

**Table 1:** Parameters used to run the simulation of Tin (Sn) based perovskite solar cells.

Parameters	ZnO	TiO_2_	SnS_2_	Zn_2_SnO_4_	CH_3_NH_3_SnI_3_	Spiro OMeTAD
Thickness (nm)	100–500	100	100	100	100–1400	100
Bandgap (eV)	3.4	3	2.24	3.35	1.30	3.2
Electron affinity (eV)	4.3	4	4.24	4.5	4.2	2.1
Dielectric permittivity	9	9	10	9	10	3
CB Effective density of states (1/cm^3^)	2 × 10^18^	2.2 × 10^18^	2.2 × 10^18^	2.2 × 10^18^	1 × 10^18^	2.5 × 10^18^
VB Effective density of sates (1/cm^3^)	1.8 × 10^20^	1.9 × 10^18^	1.8 × 10^19^	1.8 × 10^19^	1.0 × 10^18^	1.8 × 10^19^
Electron thermal velocity (cm/s)	1 × 10^7^	1 × 10^7^	1 × 10^7^	1 × 10^7^	1 × 10^7^	1 × 10^7^
Hole thermal velocity (cm/s)	1 × 10^7^	1 × 10^7^	1 × 10^7^	1 × 10^7^	1 × 10^7^	1 × 10^7^
Electron mobility (cm^2^/Vs)	1 × 10^2^	2	50	32	1.6	2 × 10^−4^
Hole mobility (cm^2^/Vs)	2.5	1	50	3	1.6	2 × 10^−4^
Donor density N_D_ (1/cm^3^)	1 × 10^19^	1 × 10^18^	1 × 10^17^	1 × 10^19^	0	0
Acceptor desity N_A_ (1/cm^3^)	0	0	0	0	3.2 × 10^15^	1.0 × 10^20^

**Table 2:** Simulation parameters used for back and front contact.

Parameters	Front Contact	Back Contact
Surface recombination velocity of electrons (cm/s)	1 × 10^7^	1 × 10^5^
Surface recombination velocity of holes (cm/s)	1 × 10^5^	1 × 10^7^
Metal work function (eV)	4.3000	5.5000
Majority carrier barrier height relative to Ef (eV)	4.3000	−0.2000
Majority carrier barrier height relative to Ev (eV	0.2796	−0.0912

**Table 3:** Variation of carrier diffusion length for the corresponding absorber defect density.

Defect density (cm^−3^)	10^14^	10^15^	10^16^	10^17^
Diffusion length (nm)	2000	750	500	320

**Table 4:** Influence of different back contact on the device performance.

	Ag	Fe	Cu	Au	Ni	Pt
Metal work function (Ev)	4.74	4.81	5.00	5.10	5.50	5.70
Efficiency (%)	9.98	11.53	15.38	15.75	15.77	15.77
Fill Factor	41.33	47.09	61.2	62.56	62.61	62.61
V_oc_ (V)	0.8311	0.8311	0.8322	0.8319	0.8319	0.8319
J_sc_ (mA cm^−2^)	29.057	29.457	30.2032	30.2703	30.2729	30.2729

**Table 5:** Device performance with different ETL materials.

ETL	V_oc_ (V)	J_sc_ (mA cm^−2^)	FF	PCE (%)
Zn_2_SnO_4_	1.1857	32.301538	64.58	24.73
ZnO	1.1089	30.458087	58.08	19.62
SnS_2_	1.1075	30.398688	56.96	19.18
TiO_2_	1.1066	30.112588	56.56	18.85

## Data Availability

The data that support the findings of this study are available on request from the corresponding author, Raja Sellappan.
